# Age-Related Health Hazards in Old Patients with First-Time Referral to a Rheumatologist: A Descriptive Study

**DOI:** 10.1155/2011/823527

**Published:** 2011-12-01

**Authors:** W. van Lankveld, M. Fransen, F. van den Hoogen, Alfons den Broeder

**Affiliations:** Department of Rheumatolgy, Sint Maartenskliniek, P.O. Box 9011, 6500 GM Nijmegen, The Netherlands

## Abstract

*Objective*. To study the prevalence of generic age-related health hazards in elderly patient referred to a rheumatologist. *Methods*. Patients aged 75 or older referred to a specialized gerontorheumatological outpatient service over a period of 2 years were studied prospectively to determine the prevalence of comorbidities, a history of falls, inactivity, cognitive dysfunction, loneliness, and depression in this patient group. *Results*. A group of 154 patients were included in the study. Comorbidities were observed in 88% of the patients. At least one fall was reported in the last year by 44% of the patients; 44% of the patients reported low levels of health-enhancing physical activity. Depressed mood and loneliness were elevated in 30% and 31% of the patients, respectively. Mild or moderate cognitive impairment was observed in 13% of the patients. *Conclusion*. Patients in this study were characterized by poor physical ability, high levels of pain, and high prevalence of age-related health hazards.

## 1. Introduction

Musculoskeletal conditions are the most frequently reported disorders in the elderly in the community [1], and a large proportion of older people is confronted with disabilities, often related to one or more musculoskeletal conditions [2]. Furthermore, it is estimated that the number of older patients with musculoskeletal conditions will double in the coming decades [3]. However, diagnostics and treatment of rheumatic conditions in the elderly can pose specific problems. These problems include coexistence of multiple musculoskeletal conditions [1], and high levels of other medical conditions in the elderly [4]. In addition, age-related physiological and psychological changes may cause both physical and psychological health hazards with possible negative effects on patient health status and treatment efficacy. Falls are common in the elderly, with potential detrimental effect for the quality of life of the patient and high numbers of fall-related hospital admissions [5]. Inactivity, or lack of health-enhancing physical exercise increases with age. Inactivity is related to survival and function in the elderly [6]. Age may also have an effect on cognitive, social, and psychological function. Cognitive function deteriorates with age in some patient, with negative effects on general health [7]. Both loneliness [8] and depression [9] are frequently observed in the elderly and may have negative consequences for the elderly patient's physical and psychological functioning. These age-related health hazards might have an effect on both health status and treatment outcome, thus warranting additional attention. Therefore, there seems a need for specialised care for elderly patients with complex rheumatologic conditions [10].

To meet these needs the gerontorheumatology outpatient service (GOS) was developed. The aim of this service is to improve treatment for the elderly patients with a first-time referral to the rheumatologist. A pilot study suggests that this service may help improve treatment of this patient group [11, 12]. The GOS addresses a wider scope of health-related variables compared to those addressed in routine rheumatology. Pain, fatigue, physical function limitations, and comorbidity are routinely assessed in rheumatology examination. In additional to these variables, the GOS addresses the aforementioned age-related health hazards that are likely to have an impact on health status and treatment.

However, as yet little is known about the frequency of these age-related health hazards in the elderly patient who presented to the rheumatologist. An important way to further improve the care for this patient group is to identify those factors that negatively effect health status, or hamper optimal treatment within this patient population. Knowing which factors are most frequently observed in the target group will help decide what additional interventions need to be developed to target these treatment needs.

Therefore, in this prospective descriptive cohort study patients with musculoskeletal conditions referred to the gerontorheumatological outpatient service over a period of 2 years were studied to determine the prevalence of comorbidities, a history of falls, inactivity, cognitive dysfunction, loneliness, and depression in this patient group.

## 2. Material and Methods

### 2.1. Study Design and Patient Recruitment

In this prospective cohort study between November 2007 and June 2009 all patients aged 75 or older with a first referral to the rheumatology outpatient service of the Sint Maartenskliniek in Nijmegen, The Netherlands, were considered for inclusion. Due to the restriction in time for the consulting rheumatologist and nurse specialist, the number of patients seen in the GOS was limited to three every week. Patients were scheduled for the GOS within the next 6 weeks. When all scheduled places were occupied, the remaining patients were attended by another rheumatologist for usual care. Patients were selected for the GOS solely based on age, and regardless of disease duration, the seriousness of their condition, recent surgery, or comorbidity.

Patients referred to the GOS were scheduled for a dual appointment at the rheumatologist and a specialized nurse. The rheumatologist assesses disease and impairment variables, the specialized nurse focuses on the patient's functioning in activities of daily life. Directly following the dual appointment, the rheumatologist and nurse decide on a further course of action, tailored to the individual's health problems and possibilities.

Three weeks prior to their appointment at the clinic, all patients were contacted by phone by the specialized nurse. Patients were asked if they were able and willing to complete a questionnaire at home prior to their visit to the clinic. It was explained to the patients that the aim of the questionnaire was to assess health status information to be used during the GOS. The questionnaires assessed demographic variables, functional ability, loneliness, depressed mood, and treatment need. Time needed to complete the questionnaire was 20 minutes, and for patients unable to complete the questionnaire at home, help was available.

The study was conducted in accordance with the Helsinki Declaration. After consulting the Local Ethics Commission, it was decided that no formal ethical approval was needed.

### 2.2. Measurements

#### 2.2.1. Patient Characteristics

Sociodemographic data were provided by the patient: gender, age (years), living status (living with spouse or alone, either unmarried, divorced, widowed), number of children, and living situation (independent or in a nursing home).

#### 2.2.2. Functional Ability

The Health Assessment Questionnaire Disability Index (HAQ-DI) was used to assess functional ability. The HAQ-DI is developed to measure function in patients with RA and is widely used in rheumatology [13]. The eight categories assessed by the Disability Index are (1) dressing and grooming, (2) arising, (3) eating, (4) walking, (5) hygiene, (6) reach, (7) grip, and (8) common daily activities. Higher scores depict higher levels of disability. Additional questions are used to assess the need for help in each category and the use of devices. Calculating the mean score for all 8 categories results in the HAQ-DI score ranging from 0 (no disability) to 3 (severe disability). In this study, the Dutch version of the HAQ-DI was used [14] which is a robust measure of physical disability [15].

*Pain* was assessed using a Visual Analogue Scale (VAS) ranging from 0 (no pain) to 100 (worst pain imaginable).

*Fatigue* was assessed using a VAS ranging from 0 (no fatigue) to 100 (worst fatigue).

#### 2.2.3. Comorbidity

The Modified Cumulative Illness Rating Scale (MCIRS) is used by the rheumatologist as a tool to indicate comorbidity [16]. It measures chronic medical illness burden while taking into account the severity of chronic diseases. On 14 diagnostic groups impairment can be rated on a 1 (no impairment) to 5 (life threatening). Summation results in a total score with a range from 14 to 70. The MCIRS is a valid indicator for health status in the geriatric residential population [17].

#### 2.2.4. Treatment Needs

Patients also indicated their treatment needs (or expectations) using item 60 of the Arthritis Impact Measurement Scales (AIMS2) [18]. In the original questionnaire patients were asked to list three items (no more or less). In this study no limit was set on the number of items patients could choose.

#### 2.2.5. Number of Falls

The patients was asked if she/he had fallen in the last year and about the frequency of falls by the nurse practitioner. The total number of falls was registered.

#### 2.2.6. Physical Activity

An adapted version of the Short Questionnaire to Assess Health-Enhancing Physical Activity (SQUASH) was used to assess physical activity. The original SQUASH measures commuting activities, leisure-time activities including sports, household activities, and activities at work and school. The SQUASH is considered to be a reliable instrument to assess patient health-enhancing activities [19]. For this study, the SQUASH was adapted for the elderly. Only the leisure time activities, sport, and household activities are assessed. For each activity the respondent is asked to rate the average number of days per week an activity is engaged in, and the average time per day. Based on the Dutch norms for people older than 50 years, health-enhancing behaviour is defined as 5 or more days of moderate intensive physical activity during 30 minutes or more each week [20]. The Dutch norms are in accordance with international guidelines [21]. 

*Cognitive functioning *was assessed by the nurse practitioner using the Mini-Mental State Examination (MMSE) [22]. The MMSE is a brief 30-point questionnaire test that is used to screen for cognitive impairment. The test covers arithmetic, memory, and orientation functions. A score above 23 points is considered normal cognitive functioning. Scores equal to or lower than 23 points are indicative of cognitive impairments.

*Loneliness *was assessed using the Loneliness scale [23, 24]. The scale was developed and is validated in the elderly population. The scale consists of 11 dichotomised items, six are formulated negatively and five are formulated positively. Positive items are recoded and items are summed resulting in a total score ranging from 0 (not lonely) to 11 (extreme lonely). Based on the scale score patients can be defined as not lonely (0–2), moderately lonely (3–8), severely lonely (9-10), and very severely lonely (11) [25].

*Depression *was assessed using the Dutch version of the Geriatric Depression Scale (GDS). The scale was developed to measure depression in the elderly [26–28]. The scale is shown to be valid and reliable in the elderly population. The scale consists of 15 dichotomised items, 9 are formulated negatively, and 5 are formulated positively. Positive items are recoded and items are summed resulting in a total score ranging from 0 (not depressed) to 15 (extremely depressed). Based on the scale score patients can be defined as not depressed (0–4), mildly depressed (5–7), moderately depressed (8–11), and severely depressed (12–15) [29].

### 2.3. Statistical Analyses

Categorical data were described as numbers and percentages. Continuous variables were described as means and standard deviation (SD). Differences between groups were calculated using chi-square for categorical variables, and *T*-test or the Kolgomorov-Smirnov test for differences between groups for continuous variables depending on distribution. Associations between variables are expressed in Pearson or Spearman correlation, depending on distribution. Based on the falls reported, the fall incidence (FI) rate is computed. FI rate is computed by dividing the total number of falls in the sample by the years of observation. In this study the years of observation are equal to the number of valid answers. To determine the prevalence of risk factors in the sample each of the measures was dichotomised (present/not present). Based on a priori defined cutoff scores on these instruments, the prevalence of risk factors in this sample was determined. The number of missing values were less than 5% for each variable. Missing values were substituted by the average scores on that variable. *P* values less than 0.05 were considered statistically significant in the analyses 95% confidence intervals are given when appropriate. All analyses were conducted using STATA [30].

## 3. Results

175 individuals of a total of 319 first referrals of elderly patients to the department of rheumatology of the Sint Maartenskliniek were seen at the gerontorheumatology outpatient service. In a total of 154 of the 175 cases records are complete and only these cases were used in the analysis. Sociodemographic variables and physical functioning indicators are given in Table 1.

The sample is characterised by low levels of physical ability and high levels of pain. Woman reported poorer functional ability compared to man (average scores is 1.5 and 1.1, resp., *T* = 2.7, *P* < .05), and functional ability was associated with higher age (*r* = 0.30; *P* < .001). Pain was unrelated to gender and age. Fatigue was unrelated to age in his sample, but women reported higher levels of fatigue compared to men (average score of 49.4 and 24.2, resp., *D* = 0.4, *P* < .0001). In Table 2 the frequency of reported rheumatic diagnosis is reported.

The most frequently reported rheumatic diagnosis is osteoarthritis. In most patients 1 rheumatic disease was diagnosed (*n* = 87). However, in 53 persons 2 diagnoses were observed and in 11 persons three, and in three patients no rheumatic diagnoses was observed.

Treatment needs as indicated by the patient in descending order were (1) pain in joints (86%), (2) movement (64%), (3) walking and bending (64%), (4) hand and finger function (57%), (5) arm function (56%), (6) social activities (56%), (7) family support (48%), (8) hygiene (42%), (9) household activities (40%), (10) mood (36%), and (11) stress (33%). On average patients indicated 5.8 (SD = 2.9) treatment needs. Age and gender were unrelated to number of treatment needs.

Prevalence of age-related health hazards.

Table 3 gives mean score (standard deviation) on the MCIRS scale, fall incidence rate (FI), and mean scores of days of health-enhancing physical activity, cognitive function, loneliness, and depression. In addition, the prevalence (%) of age-related health hazards in the sample was computed. The presence of one or more comorbidities is considered a age-related health hazard. In a similar way, one reported fall in the last year is considered a health hazards as it predicts future falling [31].

Mean burden of comorbidity assessed with the MCIRS (range 14–70) was 18.9 (SD = 2.6). The mean number of comorbidities was 2.3 (SD = 1.6; range 0–7). Only 12% of the patients were without comorbidity. On average patients take 4.3 different medications (SD = 2.0; range = 0–9).

A complete overview of type and frequency of comorbidities is given in Table 4.

Average scores on the MCIRS excluding musculoskeletal conditions was 15.7 (SD = 2.8). This adjusted MCRI score was used in further analysis.

Table 3 shows that 68 patients or 44% reported one or more falls in the last year. Most patients (*n* = 43) reported one fall, 2 falls were reported by 13 patients, and 3 or more were reported by 12 patients. Fall incidence (FI) rate is 0.75 (total number of falls/number of years of assessment = 115/154). Mean number of days being involved in health-promoting physical activities assessed with the SQUASH was 5.9 days a week with 56% of the patients meeting the criteria for health-enhancing exercise. Cognitive impairment as indicated by MMSE scores <24 were reported in 13% of the observations. No patients with severe cognitive impairment were included (MMSE <10). In 30% of the patients some level of loneliness was reported, with 27% of the patients reporting moderate levels of loneliness, and only 3% of the patients reporting severe or extreme loneliness. Depression was elevated in 31% of the patients, with 19% reporting mild levels of depression, 11% moderate levels of depression, and 2% severe depression. Most patients were not depressed according to GDS score (70% within the range of 0–4). The mean number of health hazards in this sample was 2.5 (SD 1.4). In 7 patients (4%) there was no health hazard measured, and in 33 (21%) only one hazard. The remaining patients had 2 (31%) or more health hazards (43%).

Next spearman's correlations were computed between health status indicators (physical function, pain, and fatigue) and age-related health hazards. Results are depicted in Table 5. Health status indicators were unrelated to falls and cognitive functioning, and showed low to moderate relations with the remaining health hazards. The second part of Table 5 depicts the associations between the health hazards. Low to moderate intercorrelations were observed.

## 4. Discussion

Age-related health hazards are frequently reported in this sample of patients. Nearly all patients suffered from one or more comorbidities. Additionally, many patients reported a fall in the last year, low levels of health-enhancing physical activity, and elevated levels of depression.

Patients referred to the gerontorheumatology outpatient services were characterised by high levels of functional ability impairment and pain. Functional ability in this sample was poor compared with average scores reported in a random sample of rheumatoid arthritis (RA) patients from an outpatient clinic. The HAQ score reported in this sample of RA patients using DMARDS was 0.93 (SD = 0.63) [32]. The difference between means observed in both groups is −0.52 (95% CI = −0.657 to −0.382). Average pain intensity was equal to pain intensity reported in patients accepted for treatment at a multidimensional pain treatment centre [33]. Fatigue in this sample is on a similar level to fatigue reported in an outpatient sample of patients with RA [34].

The most frequently observed health hazard in this sample was comorbidities. Falls and inactivity were both observed in a substantial proportion of patients. Fall incidence is high in this sample compared to the healthy elderly. Fall incidence assessed in 1100 community dwelling elderly, with an average age of 71, was 0.46 [35]. Mean scores of loneliness in this sample do not differ from levels of loneliness reported in a general geriatric population [24]. The prevalence of depression in this study is high compared to community-based-population elderly. For instance, one study using the same GDS-15 indication of depression, reported a prevalence of 15.4%, compared to the 31% observed in this study [36]. On the other hand, much higher levels of depression are found in housebound elderly with rheumatologic conditions, with one study reporting depression in 51.4% of the participants [37]. However, patients that are homebound due to their rheumatic condition are not representative for elderly patients with a first-time referral to a rheumatologist. The majority of patients in our study reported no feelings of loneliness or depression, and the number of patients with severe loneliness or depression was small. Severe cognitive impairment is also rare in this sample. In 13% of the patients mild to moderate levels of cognitive impairment were reported. Population-based prevalence of mild cognitive impairment in the USA is 22.2% for people aged 71 years or older [7]. It is possible that patients with cognitive dysfunction are referred to other specialists, as existing special care for the elderly is primarily focused on patients with cognitive problems [38]. Low to moderate intercorrelations were observed between health hazard indicators. Because the health hazards are relatively independent from each other, health hazards should be assessed independently. Surprisingly, the frequency of falls is unrelated to any of the physical function variables in this study.

Some limitation of the study have to be taken into consideration. An important limitation is the assessment of falls and activity as used in this study. Relying on retrospective self-reported falls is likely to result in an underestimation of the real number of falls [39]. Using prospective methods of fall registration is more time consuming but is likely to result in higher prevalence of falls. Activity assessment in this study used the SQUASH. The original SQAUSH is a valid and reliable instrument that can be used as a self-report questionnaire or a structured interview [19]. However, we have used an adapted version with a restricted number of items. Therefore, comparing the results with other studies is impossible. In addition, it is unclear whether a structured interview as used in the SQUASH has caused an answering bias. This is particularly true for those patients that came to the GOS accompanied by a relative, as is often the case in the elderly. Furthermore, no data are available on disease duration and length of treatment by the general practitioner before the patient was referred to the rheumatologist. A final limitation of the study is the definition of age-related health hazards. It is not always possible to distinguish age-related health hazards from consequences of the rheumatic disease itself. For instance, studies in RA have reported elevated levels of comorbidity [40], falls [41], and depression [42] suggesting that these hazards might be associated with the disease itself, as well as with age.

## 5. Conclusion

The current study underlines the high frequency of age-related health hazards in the elderly with musculoskeletal conditions and a first-time referral to a rheumatologist. Additional interventions targeted at these health hazards might improve health care for this patient group. Effective treatment is now available to reduce the number of falls in both the elderly [43] and in patients with osteoporosis [44], improve physical exercise for the older patient with arthritis [45], improve social networking in the elderly [46], and improve depressed feelings in elderly patients with chronic diseases [47]. Given the frequency of falls in this population and increased fracture risk in patients with rheumatic disorders [48], the development of fall-prevention programmes is highly recommended. However, more research is needed into the effect of these interventions for this particular patient group.

## Figures and Tables

**Table 1:** Means (standard deviations) and percentages of sociodemographic variables, function, pain, and fatigue (*N* = 154).

		Mean	SD
Sociodemographic variables			
Age		79.2 years	5.1
Sex (% female)		79%	
Married/cohabiting		43%	
Community dwelling		94%	
Physical functioning			
Functional ability	HAQ-DI	1.46	0.8
Pain	VAS	62.5	23
Fatigue	VAS	44.0	31

**Table 2:** Rheumatic diagnosis (*n* = 154).

Osteoarthritis	76	49%
Spondylosis	48	31%
Rheumatoid arthritis	26	17%
Arthritis otherwise defined	18	12%
Gout	9	6%
Chodrocalcinosis	18	12%
Osteoporosis	2	1%
Shoulder problem	10	6%
Polymyalgia rheumatica	5	3%
Soft tissue	2	1%
Carpal tunnel syndrome	3	2%
Others	9	6%

**Table 3:** Age-related health hazards.

		Mean (SD)	Prevalence
Comorbidities	MCIRS	18.9 (2.6)	88%^1^
Falls	FI^2^	0.77	44%
Physical exercise	SQUASH	5.9 (5.4)	44%
Cognitive function	MMSE	27.2 (2.9)	13%
Loneliness	LS	1.8 (2.5)	30%
Depressed mood	GDS	3.7 (3.0)	31%

^1^Presence of conditions other than musculoskeletal. ^2^Falls incidence: number of reported falls/number of years observed.

**Table 4:** Comorbidity (*n* = 154).

Cardiac	54	36%
Hypertension	61	40%
Vascular	39	25%
Respiratory	19	12%
EENT (Eye, ear, nose, and throat)	35	21%
Upper GI	21	14%
Lower GI	16	10%
Hepatic	4	3%
Kidney	4	3%
Other GU	24	16%
Neurological	27	18%
Endocrine/metabolic	32	21%
Psychiatric/behavioural	13	8%

**Table 5:** Spearman's correlation between health status indicators and age-related risk factors.

	Comorbidities	Falls	Activity	Cognitive	Loneliness	Depressed M.
*Physical function*						
HAQ-DI	.32*	.15	−.45**	.19	.26*	.55**
Pain	.07	−.07	−.23*	−.02	.03	.34**
Fatigue	.23*	−.03	−.27*	.05	.20*	.40**

*Health hazards*						
Comorbidities	—	−.04	−.31*	−.14	.29*	.36**
Falls	−.04	—	−.01	−.03	.14	.20*
Activity level	−.31*	−.01	—	.25*	−.22	−.42**
Cognitive impairment	−.14	−.03	.25*	—	.18*	−.27*
Loneliness	.29*	.14	−.22	.18*	—	.44**
Depression	.36**	.20*	−.42**	−.27*	.44**	—

*P < .05; **P < .01.
